# Bacteroides fragilis enterotoxin gene sequences in patients with inflammatory bowel disease.

**DOI:** 10.3201/eid0602.000210

**Published:** 2000

**Authors:** T P Prindiville, R A Sheikh, S H Cohen, Y J Tang, M C Cantrell, J Silva

**Affiliations:** University of California, Davis Medical Center, Sacramento, CA, USA.

## Abstract

We identified enterotoxigenic Bacteroides fragilis in stool specimens of patients with inflammatory bowel disease and other gastrointestinal disorders. The organism was detected in 11 (13.2%) of 83 patients with inflammatory bowel disease. Of 57 patients with active disease, 19.3% were toxin positive; none of those with inactive disease had specimens positive for enterotoxigenic Bacteroides fragilis gene sequences.


### *Bacteroides fragilis* Enterotoxin Gene Sequences in Patients with Inflammatory Bowel Disease

**Thomas P. Prindiville, Rafik A. Sheikh, Stuart H. Cohen, Yajarayma J. Tang, Mary C. Cantrell, and Joseph Silva, Jr.**
University of California, Davis Medical Center, Sacramento, California, USA

We identified enterotoxigenic *Bacteroides fragilis* in stool specimens of patients with inflammatory bowel disease and other gastrointestinal disorders. The organism was detected in 11 (13.2%) of 83 patients with inflammatory bowel disease. Of 57 patients with active disease, 19.3% were toxin positive; none of those with inactive disease had specimens positive for enterotoxigenic *Bacteroides fragilis* gene sequences.

*Bacteroides fragilis*, a gram-negative rod, constitutes 1% to 2% of the normal colonic bacterial microflora in humans (1,2). It is frequently associated with extraintestinal infections such as abscesses and soft tissue infections, as well as diarrheal diseases in animals and humans (3-6). Enterotoxigenic *B. fragilis* (ETBF) is an emerging enteric pathogen associated with diarrheal diseases in children, adults, and animals (4,7-9). The pathogen is unusual in children during the first year of life, but diarrhea associated with it is common in children 1 to 5 years of age, which suggests early acquisition of the organism and maternal protection. The pathogenicity of *B. fragilis* is related to its production of a potent enterotoxin, a zinc-containing metalloprotease with a molecular weight of 20,000 (10). The enterotoxin is tight-junction specific; causes rounding, swelling, and pyknosis of cultured enterocytes; and induces a fluid response in ligated intestinal loops and a cytotoxic response in the HT-29 colon cell line (7).

Ulcerative colitis and Crohn disease are inflammatory diseases of the gastrointestinal tract characterized by spontaneous remissions and relapses. Many microbial pathogens, particularly *Mycobacterium paratuberculosis*, paramyxoviruses, and *Listeria monocytogenes* have been implicated in the etiology of inflammatory bowel disease (IBD) (11). In addition, enteric pathogens such as *Campylobacter jejuni*, *Salmonella*, *Shigella*, *Yersinia*, and *Escherichia coli* have also been associated with relapses of IBD (12). We investigated the prevalence of ETBF in 83 patients with idiopathic IBD, in 18 patients with routine culture-negative diarrhea, and in a control population of 69 outpatients (Table 1).

Address for correspondence: Stuart H. Cohen, Department of Internal Medicine, Division of Infectious Diseases, 4150 V Street, Patient Services and Support Building, Suite 500, Sacramento, CA 95817, USA; fax: 916-734-7766; e-mail: stcohen@ucdavis.edu.

Table 1. Demographic characteristics of patients with inflammatory bowel disease (IBD)

| | Patient group | | |
|---|---|---|---|
| Patient characteristics | IBD (n = 83) | Diarrhea patients (n = 18) | Controls (n=69) |
| Sex | | | |
| M | 38 | 4 | 40 |
| F | 45 | 14 | 29 |
| Age (yrs) | 10-80 | 20-75 | 40-72 |
| Mean = | 45.5 | 45.6 | 58.2 |
| Duration of disease (yrs) | | | |
| UC | 1-13 (5.6) | 1-6 (2.6) | NA |
| CD | 1-20 (10.5) | | |
| Therapeutic treatment | | | |
| 5-ASA | 58 | 0 | 0 |
| 5-ASA+Steroids | 35 | 0 | 0 |
| 6-MP/ Azathioprine | 20 | 0 | 0 |
| MTX | 1 | 0 | 0 |
| Antibiotics | 1 | 1 | 0 |
| Antidiarrheals | 1 | 3 | 8 |

### The Study

The study protocol was approved by the Human Subjects Research Review Committee of the University of California, Davis. All samples were collected after informed consent was obtained. Of the 83 patients in the IBD group, 60 had Crohn disease, and 23 had ulcerative colitis (Table 2). Active disease was present in 68.6% of these patients on endoscopy (20 ulcerative colitis and 37 Crohn disease patients) in the form of mucosal erythema and ulceration. In the miscellaneous diarrhea group, 10 patients had irritable bowel syndrome; however, no mucosal erythema, edema, or ulceration was observed. None of the control patients had a history of diarrhea, and no erythema or ulceration was observed.

Table 2. *Bacteroides fragilis* enterotoxin gene amplification products in patients with inflammatory bowel disease (IBD)

| Patient group (no.) | ETBF[a] Gene (+) | p value[b] |
|---|---|---|
| IBD (83) | 11 (13.25%) | 0.023 |
| Active (57) | 11 (19.3%) | 0.0026 |
| Inactive (26) | 0 | |
| Crohn disease (60) | 5 | |
| Active (37) | 5 | |
| Inactive (23) | 0 | |
| Ulcerative colitis (23) | 6 | |
| Active (20) | 6 | |
| Inactive (3) | 0 | |
| Diarrhea (18) | 5 (27.8%) | 0.0005 |
| Control (69) | 2 (2.89%) | |

[a]ETBF = enterotoxigenic *Bacteroides fragilis*
[b]p value in relation to control group

Fecal specimens from the IBD group were collected endoscopically from patients undergoing colonoscopy or flexible sigmoidoscopy for evaluation of symptoms of diarrhea or abdominal pain. The fecal specimens from the 18 diarrhea patients with negative routine stool cultures and the 69 controls were collected by endoscopy.

Fecal specimens were cultured for *B. fragilis* in the selective medium Bacteroides Bile Esculin agar. Positive cultures were identified by using the RapID ANA II Panel (REMEL, Inc, Lenexa, KS). Plates were incubated anaerobically at 37°C for 48 hours. The presence of *B. fragilis* enterotoxin in the isolates was detected in the HT-29 colon cell line (13,14). HT-29 cells were grown and maintained in RPMI medium with glutamine (Gibco, Life Technologies, Inc., Grand Island, NY) supplemented with penicillin (100 IU/ml), streptomycin (100 µg/ml) (Sigma, Saint Louis, MO), and heat-inactivated fetal bovine serum (Hyclone Laboratories, Inc., Logan, UT) at 12% in 25-ml flasks at 37°C in 5% $CO_2$. For the cytotoxicity assay, HT-29 cells were suspended in 20 ml of medium for each plate, and 180 µl/well were placed into 96-well tissue culture plates (Corning Glass Works, Corning, NY). The cells were allowed to attach and grow for 2 to 3 days. Supernatants from *B. fragilis* isolates grown on Brain Heart Infusion Broth were filtered through a 0.45-µm Acrodisk syringe filter (Gelman Sciences, Ann Arbor, MI), and 20-µl serial dilutions were placed into the wells in duplicate. The plates were incubated at 37°C for 3 to 4 hours under 5% $CO_2$ and examined for typical cytopathic changes. Cultures were considered positive for *B. fragilis* enterotoxin if a visible cytopathic effect was neutralized by specific antiserum. The highest dilution of the culture supernatant producing cytopathic changes in at least 50% of the cells after 3 to 4 hours of incubation was considered the cytotoxic titer. For the neutralization assay, dilutions of 1:25 anti-enterotoxin rabbit antiserum in phosphate-buffered saline were mixed with culture supernatants positive for enteroxin. After incubation for 30 minutes at 37°C, 20 µl of each mixture was inoculated into HT-29 cells as in the cytotoxicity assay. Neutralization was indicated by the lack of cytotoxic effect.

DNA was extracted from the fecal specimens and amplified by using specific primers (14) to detect *B. fragilis* enterotoxin gene sequences.[1] Chi-square was used to determine statistical significance.

[1]A 100-mg sample of stool was suspended in 400 µl of TES buffer (50 mM Tris [pH 8], 5 mM EDTA, 50 mM NaCl) and centrifuged at 1,000 x g for 3 min to remove large particles. The supernatant was then centrifuged at 5,000 x g for 7 min. The pellet was washed once in 200 µl of TES, and centrifuged at 5,000 x g for 3 min, and the supernatant was discarded. The pellet was suspended in 100 µl of sterile $H_2O$ and boiled for 10 min, then centrifuged at 1,000 x g for 2 min and the supernatant containing the DNA extracted twice with phenol: chloroform: isoamyl alcohol (25:24:1) and precipitated with ethanol. The sequences of the primers and probes used and PCR conditions were as described (14). Amplification with the outer primers RS-3 (5' TGAAGTTAGTGCCCAGATGCAGG 3') and RS-4 (5' GCTCAGCGCCCAGTATA TGACC 3') yielded a 367-bp product. Amplification of this product with the inner primers RS-1 (5' TGCGGCGAACTCGGTTAATGC 3') and RS-2 (5'AGCTGGGTTGTAGACATCCCACTGG' 3') amplified a 290-bp product. The reaction mixtures were prepared in 1X PCR buffer (50 mM KCl, 20 mM Tris HCl, 2.5 mM $MgCl_2$, 100 µg bovine serum albumin per ml [pH 8.4]) and contained per reaction 20 pmol of the respective primers, 0.1 mM concentrations each of 2'-deoxynucleoside 5'-triphosphate, 2 U of recombinant DNA polymerase (rTaq) (Perkin Elmer, Norwalk, CT), and 10 µl purified fecal DNA. The final reaction volume was adjusted to 100 µl with sterile deonized water. The PCR profile included a denaturing step at 95°C for 30 sec, followed by a 60°C annealing step for 30 sec, with extension at 72°C for 30 sec. The outer PCR was performed for 35 cycles in a thermal cycler (MJ Research). Amplification with the inner primers was done for 30 cycles. Negative controls included a blank containing all PCR reagents with no DNA. As control for amplifiable DNA in the stool specimens, primers targeting the 16S rRNA gene of enteric bacteria were used as described by Kato et al. (15).

ETBF was cultured from four patients with IBD and two patients with diarrhea. However, *B. fragilis* enterotoxin gene sequences were detected in the stools of 11 (13.25%) of 83 patients with idiopathic IBD (five with Crohn disease and six with ulcerative colitis). All 11 patients positive for ETBF had active disease by endoscopic and histologic tests (Table 2). No ETBF was found in patients with inactive disease. The Crohn disease patients who were positive for enterotoxin gene sequences had superficial mucosal disease. In the control group, 2 (2.9%) of 69 patients were positive for *B. fragilis* enterotoxin gene sequences. Enterotoxin amplification products were also detected in the specimens of 5 (27.7%) of 18 patients with diarrhea due to miscellaneous causes (Table 2). All the specimens were positive when amplified with primers specific for the 16S rRNA gene of enteric bacteria.

### Conclusions

The normal colonic microflora of humans is a complex ecosystem of approximately 500 species of aerobic and anaerobic microorganisms. Although the gut of the newborn infant is sterile, *Bacteroides* species—the predominant anaerobic constituent of the colonic flora—appear at approximately 10 days, are established by 2 weeks, and usually remain constant lifelong (1,2). In breast-fed infants, *Bifidobacterium* are the predominant population, and *Bacteroides* group organisms remain undetectable. However, after weaning, the *Bacteroides* group organisms increase and *Bifidobacterium* organisms decrease substantially (16).

The exact etiology of idiopathic IBD is still unknown, although a potential role for infectious agents or toxins that may stimulate an inflammatory response has been suggested (16,17). For example, *Peptostreptococcus*, *Coprococcus*, and *Bacteroides* sp. have been reported in patients with Crohn disease (17). A role for these microorganisms in the disease process is also suggested by the clinical responses of some patients to antibiotics (18). Observations of *B. thetaiotaomicron* in patients with ulcerative colitis (16) and *B. vulgatus* in guinea pigs with experimentally induced ulcerative colitis (19) suggest that microorganisms may influence the development or maintenance of intestinal inflammation in IBD.

In this study, *B. fragilis* enterotoxin gene sequences were detected by nested polymerase chain reaction (PCR) in the stools of 13.2% of patients with inflammatory bowel disease and 2.9% controls. The low recovery of ETBF in culture may be due to the length of time from specimen collection to processing in the laboratory (most specimens were kept frozen for at least 2 weeks before culturing). Similar results have been reported by Sack et al. (9), who found a marked reduction in the recovery of *B. fragilis* with time. Amplification of enterotoxin gene directly in the stools of these patients appears to be a more sensitive detection method. In a previous study (14), we found 100% correlation between PCR and enterotoxin production in isolates; we therefore did not routinely perform the HT-29 cell assay in specimens of these patients.

In the IBD group, all the patients positive for ETBF had active disease, which suggests an association with disease activation or flare-up. ETBF was also found in patients with ulcerative proctitis, collagenous colitis, and microscopic colitis. In patients with Crohn disease, ETBF was usually seen in the colonic superficial inflammatory disease type. The presence of ETBF in IBD patients may represent alterations of endogenous bacterial flora, which may be related to either the etiology or flare-up of the disease or both. Colonization with ETBF may be acquired early in life or may be a de novo infection related to flare-ups of the disease.

### Acknowledgment

The authors thank David M. Lyerly for providing the anti-enterotoxin rabbit antiserum.

Dr. Prindiville is an associate professor of medicine in the Division of Gastroenterology at the University of California, Davis, California, USA.

### References

1. Moore WE, Cato EP, Holdeman LV. Some current concepts in intestinal bacteriology. Am J Clin Nutr 1978;31:S33-42.
2. Simon GL, Gorbach SL. Intestinal flora in health and disease. Gastroenterology 1984;86:174-93.
3. Gorbach SL, Barlett JG. Anaerobic Infections. N Engl J Med 1974;290:1177-84.
4. Myers LL, Firehammer BD, Shoop DS, Border MM. Bacteroides fragilis: a possible cause of acute diarrheal diseases in newborn lambs. Infect Immun 1984;44:241-4.

5. Sack RB, Myers LL, Almeido-Hill J, Shoop DS, Bradbury WC, Reid R, et al. Enterotoxigenic *Bacteroides fragilis*: epidemiologic studies of its role as a human diarrhoeal pathogen. J Diarrhoeal Dis Res 1992;10:4-9.
6. Pantosti A, Menozzi MG, Frate A, Sanfilippo L, D'Ambrosio F, Malpeli M. Detection of enterotoxigenic *Bacteroides fragilis* and its toxin in stool samples from adults and children in Italy. Clin Infect Dis 1997;24:12-6.
7. Weikel CS, Grieco FD, Reuben J, Myers LL, Sack RB. Human colonic epithelial cells, HT-29/C1, treated with crude Bacteroides fragilis enterotoxin dramatically alter their morphology. Infect Immun 1992;60:321-7.
8. SanJoaquin VH, Griffis JC, Lee C, Sears CL. Association of *Bacteroides fragilis* with childhood diarrhea. Scand J Infect Dis 1995;27:211-5.
9. Sack RB, Albert MJ, Alam K, Neogi PK, Akbar MS. Isolation of enterotoxigenic *Bacteroides fragilis* from Bangladeshi children with diarrhea: a controlled study. J Clin Microbiol 1994;32:960-3.
10. VanTassell RL, Lyerly DM, Wilkins TD. Purification and characterization of an enterotoxin from *Bacteroides fragilis*. Infect Immun 1992;60:1343-50.
11. Sartor RB. Microbial factors in the pathogenesis of Crohn's disease, ulcerative colitis, and experimental intestinal inflammation. In: Kirsner JB, Shorter RJ, editors. Inflammatory bowel disease. 4th ed. Baltimore: Williams and Wilkins; 1995. p. 96-124.
12. Hermens DJ, Miner PB Jr. Exacerbation of ulcerative colitis [clinical reference]. Gastroenterol 1991;101:254-62.
13. Pantosti A, Cerquetti M, Colangeli R, D'Ambrosio F. Detection of intestinal and extraintestinal strains of enterotoxigenic *Bacteroides fragilis* by the HT-29 cytotoxicity assay. J Med Microbiol 1994;41:191-6.
14. Shetab R, Cohen SH, Prindiville TP, Tang YJ, Cantrell M, Rahmani D, et al. Detection of *Bacteroides fragilis* enterotoxin gene by PCR. J Clin Microbiol 1998;36:1729-32.
15. Kato N, Ou CY, Kato H, Bartley SL, Luo CC, Kilgore E, et al. Detection of toxigenic *Clostridium difficile* in stool specimens by the polymerase chain reaction. J Infect Dis 1993;167:455-8.
16. Dore J, Sghir A, Hannequart-Gramet G, Corther G, Pochart P. Design and evaluation of a 16S rRNA-targeted oligonucleotide probe for specific detection and quantitation of human faecal Bacteroides populations. System Appl Microbiol 1998;21:65-71.
17. Van de Merwe JP, Schroder AM, Wensinck F, Hazenberg MP. The obligate anaerobic faecal flora of patients with Crohn's disease and their first degree relatives. Scand J Gastroenterol 1988;23:1125-31.
18. Sartor RB. Current concepts of the etiology and pathogenesis of ulcerative colitis and Crohn's disease. Gastroenterol Clin North Am 1995;24:475-507.
19. Onderdonk AB, Cisneros RL, Bronson RT. Enhancement of experimental ulcerative colitis by immunization with *Bacteroides vulgatus*. Infect Immun 1983;42:783-8.
